# Multiplicative disadvantage of being an unmarried and inadequately insured woman living in poverty with colon cancer: historical cohort exploration in California

**DOI:** 10.1186/s12905-015-0166-5

**Published:** 2015-02-07

**Authors:** Naomi R Levitz, Sundus Haji-Jama, Tonya Munro, Kevin M Gorey, Isaac N Luginaah, Emma Bartfay, Guangyong Zou, Frances C Wright, Sindu M Kanjeekal, Caroline Hamm, Madhan K Balagurusamy, Eric J Holowaty

**Affiliations:** School of Social Work, University of Windsor, Windsor, Ontario Canada; Dalla Lana School of Public Health, University of Toronto, Toronto, Ontario Canada; School of Social Work, University of Windsor, Windsor, Canada; Department of Geography, Western University, London, Ontario Canada; Faculty of Health Sciences, University of Ontario Institute of Technology, Oshawa, Ontario Canada; Department of Epidemiology and Biostatistics, and Robarts Research Institute, Western University, London, Ontario Canada; Division of General Surgery, Sunnybrook Health Sciences Center and Departments of Surgery and Health Policy, Management and Evaluation, University of Toronto, Toronto, Ontario Canada; Medical Oncology Department, Windsor Regional Cancer Center, Windsor, Ontario Canada; Medical Oncology Department, Windsor Regional Cancer Center and School of Medicine and Dentistry, Department of Oncology, Western University, London, Canada

**Keywords:** Health insurance, Poverty, Colon cancer, Chemotherapy, Marital status, Unmarried, Women, California, United States

## Abstract

**Background:**

Many Americans diagnosed with colon cancer do not receive indicated chemotherapy. Certain unmarried women may be particularly disadvantaged. A 3-way interaction of the multiplicative disadvantages of being an unmarried and inadequately insured woman living in poverty was explored.

**Methods:**

California registry data were analyzed for 2,319 women diagnosed with stage II to IV colon cancer between 1996 and 2000 and followed until 2014. Socioeconomic data from the 2000 census classified neighborhoods as high poverty (≥30% of households poor), middle (5–29%) or low poverty (<5% poor). Primary health insurance was private, Medicare, Medicaid or none. Comparisons of chemotherapy rates used standardized rate ratios (RR). We respectively used logistic and Cox regression models to assess chemotherapy and survival.

**Results:**

A statistically significant 3-way marital status by health insurance by poverty interaction effect on chemotherapy receipt was observed. Chemotherapy rates did not differ between unmarried (39.0%) and married (39.7%) women who lived in lower poverty neighborhoods and were privately insured. But unmarried women (27.3%) were 26% less likely to receive chemotherapy than were married women (37.1%, RR = 0.74, 95% CI 0.58, 0.95) who lived in high poverty neighborhoods and were publicly insured or uninsured. When this interaction and the main effects of health insurance, poverty and chemotherapy were accounted for, survival did not differ by marital status.

**Conclusions:**

The multiplicative barrier to colon cancer care that results from being inadequately insured and living in poverty is worse for unmarried than married women. Poverty is more prevalent among unmarried women and they have fewer assets so they are probably less able to absorb the indirect and direct, but uncovered, costs of colon cancer care. There seem to be structural inequities related to the institutions of marriage, work and health care that particularly disadvantage unmarried women that policy makers ought to be cognizant of as future reforms of the American health care system are considered.

## Background

Chemotherapy is often indicated for those diagnosed with non-localized colon cancer. Survival benefits are large for many with stage III disease and smaller, but still significant for some with stage II disease [1-3]. In addition to its palliative benefits, there even seem to be some survival benefits of chemotherapy for people with stage IV colon cancer [4], yet nearly half of such people in the United States never access chemotherapy.^5^ Similar to health care in general, access to chemotherapy may be affected by a variety of causes. Chemotherapy access may be affected by social and economic characteristics such as marital status, health insurance adequacy, income and race or ethnicity [5-8]. Because colon cancer is relatively common over the life course, adjuvant chemotherapy matters in terms of both survival and quality of life and such treatment access seems sensitive to the sorts of social and policy forces that probably determine much of America’s health inequities, chemotherapy of non-localized colon cancer may be thought a sentinel health care quality indicator.

A 3-way ethnicity by health insurance by poverty interaction effect on chemotherapy receipt was recently observed among people with non-localized colon cancer in California [8]. A multiplicative barrier to chemotherapy was found suggesting that the combined effects of being inadequately insured and living in poverty were worse for African Americans than for non-Hispanic white Americans. Those investigators concluded that because African Americans are more prevalently poor, inadequately insured and have fewer assets; they are probably less able to absorb the indirect and direct, but uncovered costs of colon cancer care. Because women, particularly unmarried women may be similarly socioeconomically vulnerable, we wondered about the possibility of a 3-way marital status by health insurance by poverty interaction effect on chemotherapy among them.

United States surveys have indicated that women are much more likely to live in poverty and to be uninsured or underinsured than are men and that being unmarried seems to increase all such risks more for women than for men [9,10]. Similarly surveys suggest that married women have a higher chance of being privately insured by so-called gold or platinum health plans through employers, regardless of whether the plan is theirs or their spouses. Even when unmarried women can afford private health insurance, they seem to be at greatest risk of being covered by bronze or silver plans, which typically come with high deductible costs [11]. Seemingly more affordable because their premiums are lower than gold or platinum plans, their deductible costs of up to $2,500 may serve to limit health care access, especially among lower income beneficiaries. Otherwise, unmarried women are more likely to need to purchase additional Medicare “medigap” coverage or experience compromised Medicaid coverage with increasing out-of-pocket expenses. These expenses have been predicted under the Affordable Care Act [12]. Meanwhile, gender and marital status seem associated with colon cancer care in the United States, but the equivocally observed care gaps have tended to be small [5,13]. We think that this field may be limited by its focus on mere main effects. Previous studies found complex interaction effects of ethnicity, health insurance status and poverty on cancer care in the United States [14-17]. For example, a historical study of colon cancer care in California, including access to chemotherapy, concluded that poverty makes the disadvantaging effects of being uninsured or underinsured even worse, specifically for women. This leads us to anticipate similarly complex interactions of marital status, health insurance and poverty among women [17]. This study explores that notion with the same Californian cohort and advances the hypothesis that there is a 3-way interaction of marital status, health insurance and poverty on chemotherapy such that there is probably a multiplicative disadvantage of being an unmarried and inadequately insured woman living in poverty.

## Methods

Three thousand, three hundred women diagnosed with colon cancer between 1996 and 2000 were randomly selected from the California Cancer Registry and joined to the 2000 census by census tracts [17-20]. The original sample was then stratified by socioeconomic status: high poverty neighborhoods where 30% or more of the households were poor, mid-poverty neighborhoods where 5% to 29% were poor or low poverty neighborhoods where the prevalence of poverty was less than 5% [21,22]. This analysis excluded localized, stage I cancers for which chemotherapy is not indicated and then analyzed colon cancer care among 2,319 women with stage II to IV disease [23,24]. Primary health insurers were defined as private (included the Medicare insured with private supplemental coverage), Medicare alone, Medicaid or none. And based upon previous analyses in California, primary health insurance was defined as adequate (private) or inadequate (Medicaid, Medicare or none) [16,17,25]. Marital status at the time of diagnosis was married or unmarried (never married or previously married).

A preliminary age, stage and grade-adjusted logistic regression model found a 4-way gender by marital status by health insurance adequacy by poverty (lived in a high poverty neighborhood or not) interaction effect on binary chemotherapy receipt such that the 3-way interaction was statistically significant among women, but not men. This study’s focus is on women and statistically tests and describes the 3-way marital status by health insurance adequacy by poverty interaction effect on chemotherapy among them [26]. Odds ratios (OR) and 95% confidence intervals (CI) were estimated for main effects from maximum likelihood regression statistics. We tested the 3-way interaction effect by adding the following interaction term to the regression model: married or not × adequately insured or not × lived in a high poverty neighborhood or not (each 0 or 1).

Married study participants were younger (*M* = 67.0, *SD* = 13.0), on average, than unmarried participants (*M* = 72.0, *SD* = 14.2), *F* (1, 4,382) = 149.48, *p* < .05. Therefore, all prevalence estimates were internally age-adjusted and reported as percentages. Chemotherapy rates were also stage-adjusted to account for variances in clinical indication and prescription rates in stages II, III and IV of the disease. Standardized prevalence ratios (PR) or rate ratios (RR) were reported for critical between-group comparisons with 95% CIs derived from the Mantel-Haenszel χ^2^ test. Standardized rate differences (RD) were also used to further aid in the interpretation of practical-clinical significance. Survival analyses used similarly adjusted Cox regression models [27]. Participants were minimally followed for 13 years from the date of their diagnosis until 2014. These analyses could detect rate differences of less than 5% with 80% power at a significance level of 5% [28]. All variables except tumor grade (6.3% missing) had less than 2% missing data, none of which were confounding so missing data were imputed from full regression models. Other methodological details have been reported [17-20]. This study was reviewed and cleared by the University of Windsor research ethics board.

## Results

Age-adjusted descriptive profiles of the married (41.7%) and unmarried (58.3%) women with non-localized colon cancer are displayed in Table 1. The statistically significant comparisons of socioeconomic characteristics were congruent with existing knowledge. Unmarried women (39.3.3%) were nearly 50% more likely than married women (27.0%) to live in high poverty neighborhoods (PR = 1.46, 95% CI 1.28, 1.66) and they were about 20% more likely to be relatively inadequately insured, either uninsured to publicly-insured (60.5% vs. 49.9%, PR = 1.21, 95% CI 1.16, 1.27). Also of interest, the two groups did not differ significantly on the clinical-biological characteristics of disease stage or tumor grade.

**Table 1:** **Demographic, socioeconomic and clinical characteristics of married and unmarried women diagnosed with colon cancer in California between 1996 and 2000**

	**Married**	**No. (%)**	**Unmarried**	**No. (%)**
**Age at diagnosis,** ^*^ **y**				
<65	388	40.1	271	20.1
65–80	458	47.3	593	43.9
>80	122	12.6	487	36.0
**Neighborhood poverty prevalence,** ^**†**^ **%**				
<5	391	40.3	376	27.0
5 – 29	317	32.7	458	33.7
≥30^‡^	260	27.0	517	39.3
**Primary health insurers** ^**†**^				
Private	510	50.1	482	39.5
Medicare	364	41.6	732	46.4
Medicaid	44	3.9	68	7.0
Uninsured	50	4.4	69	7.1
**Stage at diagnosis**				
II	409	43.5	569	40.9
III	318	32.5	417	31.5
IV	241	24.0	365	27.6
**Tumor grade**				
I	72	7.8	87	6.5
II	597	65.0	793	63.6
III or IV	247	27.2	376	29.9
Missing data	52	5.4	95	7.0

*Notes*. Prevalence estimates (%) were directly age-adjusted using this study’s population of women as the standard.

^*^ *p* < .05 marital group difference (*χ* ^2^ test).

^†^ *p* < .05 age-adjusted marital group difference (Mantel-Haenszel *χ* ^2^ test).

^‡^Median annual family income for married ($23,450) and unmarried ($22,325) subsamples of women; median test [29] *χ* ^2^ (1, *N* = 777) = 4.12, *p* < .05.

As hypothesized, a statistically significant 3-way marital status by health insurance by poverty interaction was detected with a full logistic regression on chemotherapy receipt (bottom of Table 2). The 2-way interactions involving marital status were also significant, but the interaction of health insurance and poverty as well as all of the main effects were not significant. Our interpretive emphasis will be on the, most complex, 3-way interaction. As its meaning is not necessarily intuitive, the receipt of chemotherapy within its strata is practically depicted in Table 3. At the top of the table it can be seen that among women who did not live in high poverty neighborhoods and were adequately insured, chemotherapy rates did not differ significantly between the married (39.7%) and unmarried (39.0%). The bottom of the table suggests that among the multiply disadvantaged, women who were inadequately insured and living in poverty, the disadvantaging effect on chemotherapy access appears multiplicative. Among them, unmarried women (27.3%) were 26% less likely to receive chemotherapy than were married women (37.1%, RR = 0.74, 95% CI 0.58, 0.95). This apparent multiplicative disadvantage is depicted in another way in the table’s right column. The married-unmarried RD on chemotherapy receipt was 0.7% among the most advantaged group. While the RD of 9.8% among the most disadvantaged group was nearly a 15-fold multiple of that baseline difference. Such seems indicative of a large barrier to chemotherapy access that accounted well for between-group survival differences. When the main and interacting effects of chemotherapy were accounted for with a Cox regression model survival no longer differed by marital status (HR = 0.98, 95% CI 0.86, 1.12). Before accounting for chemotherapy unmarried women were at significantly greater risk of dying over the follow-up period (HR = 0.88, 95% CI 0.80, 0.98).

**Table 2:** **Logistic regression results for main effects and interactions of marital status, neighborhood poverty and primary health insurers: Predictors of chemotherapy receipt**

**Predictors**	**Odds ratio**	**95% Confidence interval**
**Nonsignificant main effects and interactions**		
Unmarried	0.72	0.36, 1.44
Neighborhood poverty prevalence ≥ 30%	0.60	0.31, 1.15
Primary health insurer was private	1.06	0.59, 1.89
Neighborhood poverty by primary health insurer	P_interaction_ = .511	
**Significant interaction effects**		
Unmarried by neighborhood poverty	P_interaction_ = .030	
Unmarried by primary health insurer	P_interaction_ = .047	
Unmarried by health insurer by poverty	P_interaction_ = .012	

*Notes*. All effects were adjusted for age, stage of disease at diagnosis, tumor grade and each other. The fit of the regression model with the 3-way interaction was significantly better than the model without it: likelihood ratio test, *χ* ^2^ (1) = 6.33, *p* < .05.

**Table 3:** **Effect of the interaction of marital status, health insurance and neighborhood poverty on chemotherapy receipt among women with stage II to IV colon cancer**

	**No.** ^*^	**Rate (%)**	**RR** ^**†**^	**(95% CI)**	**Married-unmarried chemotherapy RD**
**<30% Poor & adequately insured**					
Married	405	39.7	1.00	. . .	
Unmarried	344	39.0	0.98	(0.82, 1.17)	0.7%
**Intermediate groups** ^**‡**^					
Married	408	38.3	1.00	. . .	
Unmarried	628	34.6	0.90	(0.76, 1.07)	3.7%
**≥30% Poor & inadequately insured**					
Married	155	37.1	1.00	. . .	
Unmarried	379	27.3	0.74	(0.58, 0.95)	9.8%

*Notes*. RR = standardized rate ratio, RD = standardized rate difference, CI = confidence interval. All rates were directly age and stage-adjusted using this study’s population of women as the standard and reported as percentages (rates per 100).

^*^Number of incident colon cancer cases.

^†^A rate ratio of 1.00 was the baseline.

^‡^Women living in high poverty neighborhoods, but adequately insured or women living in less poor neighborhoods, but inadequately insured. These two groups did not differ significantly on their married-unmarried chemotherapy RDs.

## Discussion

We explored a 3-way marital status by health insurance by poverty interaction effect on the receipt of chemotherapy among women with non-localized colon cancer. More specifically, we observed an apparent multiplicative barrier to chemotherapy of being an unmarried and inadequately insured woman living in poverty. Poverty seems to worsen the disadvantaging effect of being inadequately insured, especially for unmarried women. Among disadvantaged, publicly or uninsured women living in poverty, the married-unmarried chemotherapy RD (10%) was not only significant in a statistical sense, but could also be characterized as practically significant. Conversely, among relatively advantaged, privately insured women not living in poverty, the married-unmarried chemotherapy RD was not significant. Given the importance of chemotherapy in the treatment of non-localized colon cancer this finding seems of great human significance. We also found evidence that the receipt of chemotherapy can explain the difference in survival chances between married and unmarried women.

Two-way health insurance by poverty interactions have previously been observed in studies of breast and colon cancer care [8,15-17]. The beneficial effects of health insurance were observed to be strongest in low poverty neighborhoods. It seems that the effectiveness of health insurance was positively impacted by the availability of other resources. In more affluent neighborhoods, where social and economic capital abounds, most people with cancer were more able to absorb the indirect and direct, but additional uncovered, out-of-pocket costs of cancer care. On the other hand, within high poverty neighborhoods relatively lacking in such capital reserves, health insurance programs were much less effective. People who lived in such high poverty neighborhoods may have been less able to pick-up the co-insurance costs and co-payments that are prevalent in American cancer care delivery. The 3-way marital status-health insurance-poverty interaction observed among women in this study could be thought an extension of the consistently observed 2-way health insurance inadequacy-poverty interaction. It suggests that the 2-way interaction’s disadvantaging effect is strongest for unmarried women. Their relative lack of income and assets could explain the differential effect of marital status.

Married women have more diverse sources of income than their unmarried or previously married peers and, therefore, typically have greater incomes and wealth. They also benefit from the economy of scales that attend sharing household and other expenses with a spouse. Married women have greater access to work-related fringe benefits, including private health insurance, through their own or their spouse’s employer [30,31]. We saw that even though this study’s key comparison of unmarried and married women was in high poverty neighborhoods, the depth of impoverishment among the unmarried women seemed deeper. Their annual family incomes were, typically, $1,125 less than those of married women. This difference may not seem great, but among people with such low incomes this represents approximately 5% of their total income. This could make a difference, for example, in being able to absorb the indirect costs of cancer care due to time lost from work, recuperation and transportation. It is also known that among low-income households in America, married women are three times more likely than unmarried women to own a home, again representative of their relatively greater wealth [32]. It seems that the relative lack of capital reserves operates to further potentiate the disadvantages already experienced by unmarried women who are inadequately insured and live in poverty.

### Potential limitations

First, one may wonder about the potential confounding influence of social supports beyond marital status. Diverse measures of perceived social support as well as numerous functional characteristics of social networks are well known to be associated with diverse health care processes and health outcomes over the life course [33]. Moreover, social and extended familial supports seem to provide protections across all phases of cancer care, from screening and treatment access to enhanced quality of life after treatment and survival [34-36]. For example, friends and extended family members might facilitate treatment access and even its success by filling such potentially important roles such as driving a friend to chemotherapy and caring for her after chemotherapy. But the strength of these associations as well as their interrelationships with other of this study’s central predictors seems equivocal. We are unaware of any consistent evidence that such supports beyond spousal support serve to increase the adequacy of one’s health insurance coverage. Furthermore, we have observed that even in such extremely poor neighborhoods as Mexican American barrios where social and extended familial supports tend to be much greater than in other similarly poor ethnic enclaves, the adequacy of one’s health insurance remains a critical predictor of cancer care and survival [14,15]. Surely, social supports beyond spousal support are very important in the lives of all people including those with cancer. But for reasons noted above we do not think it likely that such supports fatally confound this study’s central finding about the interaction of marital and insurance statuses and poverty.

Second, the marital status variable that was available for this analysis was limited in a few ways. First, its categories did not include cohabitation, that is, those who live together as partners, who would likely share many resources and expenses, but are not legally married. Probably quite rare among this study’s sample of women [37], seven of ten of whom were 65 years of age or older, certainly some of those we classified as unmarried may enjoy some such marriage-like benefits. Second, marital status was only assessed at diagnosis, not accounting for marital statuses over the life course. Many of those who were not married at the time of their diagnosis, for example the widowed and divorced, were previously married, some for many years. Again, they may have enjoyed some marriage-like benefits, though a wealth of research has shown that the previously married tend to be more similar to the never married than to the married in terms of their economic well-being [30]. It is clear though that both of these forms of differential misclassification bias, if they are potent at all, would operate to cause this study to underestimate the disadvantages of being an unmarried woman seeking colon cancer care.

Third, because chemotherapy is most often received as an outpatient it can be challenging for cancer registries to survey. However, we think that this study is not potently limited by incomplete information on chemotherapy for the following reasons. First, the California Cancer Registry is nearly complete on chemotherapy (82–83%) and comprehensiveness has been demonstrated not to differ significantly by marital status [38]. Second, missing chemotherapy data was very modest in this analysis and did not differ significantly between married and unmarried samples of women. Other potential limitations have been reported [17-20]. Such potential limitations make it clear that more studies are needed to confirm, or refute, the 3-way interaction that this study explored.

## Conclusions

Among the uninsured or underinsured living in poverty unmarried women seemed less likely than married women with non-localized colon cancer to receive indicated chemotherapy. Marital status, health insurance status and poverty appear to interact in such a way that the multiplicative barrier to care of being inadequately insured and poor is worst for unmarried women. They are the most inadequately insured and prevalently poor, and they hold the fewest assets, and so, are probably the least able to absorb the indirect and direct, but uncovered costs of colon cancer care. This suggests that there are structural inequities related to the institutions of marriage, work and health care that particularly disadvantage unmarried women. Policy makers and administrators ought to be cognizant of these factors as they consider future reforms of the American health care system.

## Abbreviations

CI: Confidence interval
OR: Odds ratio
PR: Prevalence ratio
RD: Rate difference
RR: Rate ratio
